# Supplementary figures and images for: An investigation into the response of the soil ecological environment to tourist disturbance in Baligou
Source: PeerJ. 2023 Sep 1;11:e15780. doi: 10.7717/peerj.15780 (PMC10476605; doi:10.7717/peerj.15780)

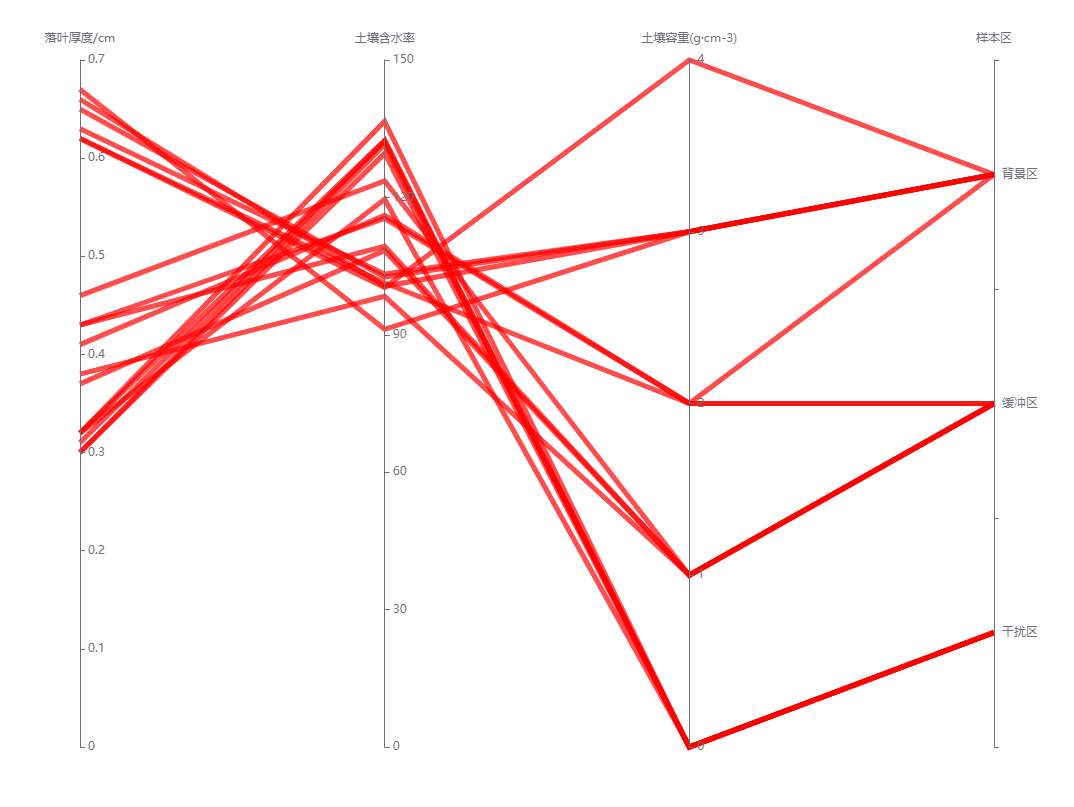

Supplement: Data S1 [file peerj-11-15780-s001.zip › Data 79893/ImageLong/ImageLong/tu1.png]

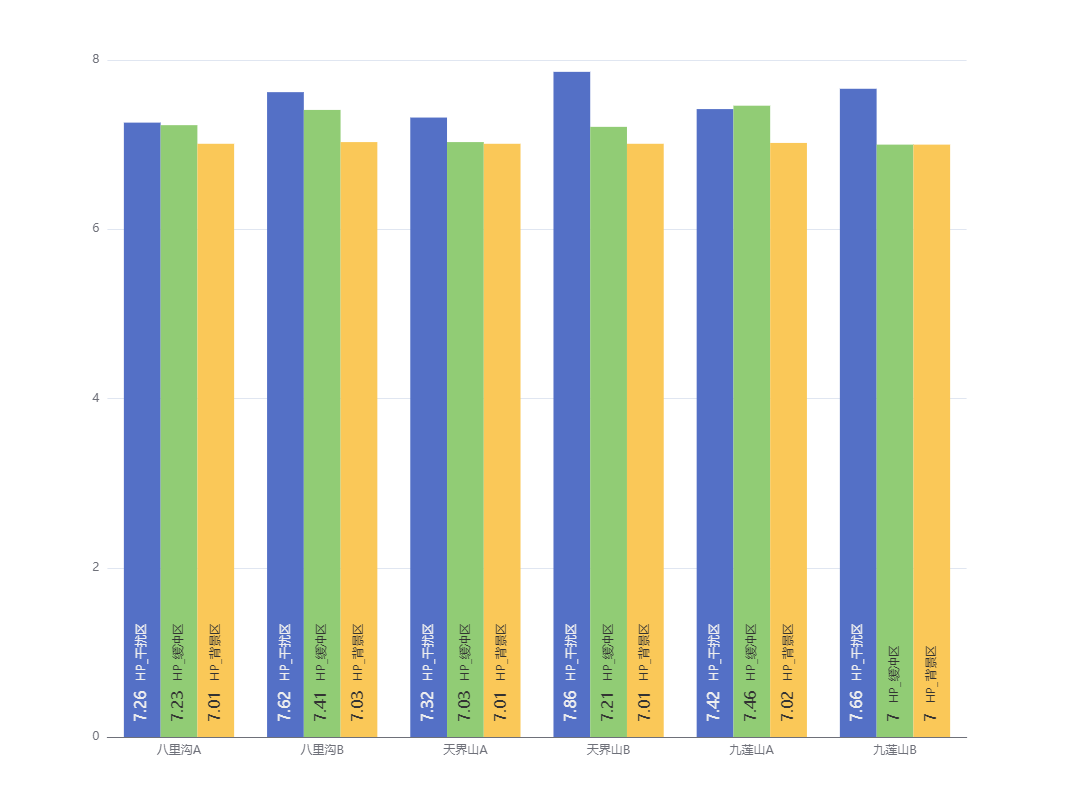

Supplement: Data S1 [file peerj-11-15780-s001.zip › Data 79893/ImageLong/ImageLong/tu2.png]

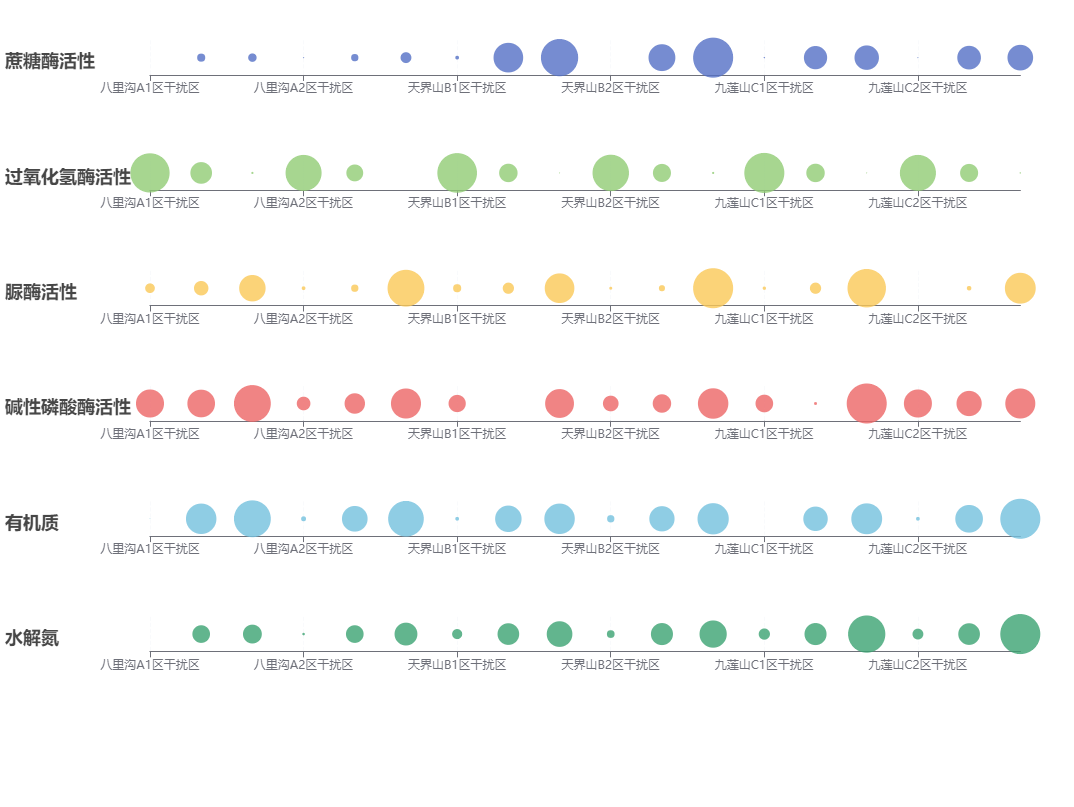

Supplement: Data S1 [file peerj-11-15780-s001.zip › Data 79893/ImageLong/ImageLong/tu3.png]

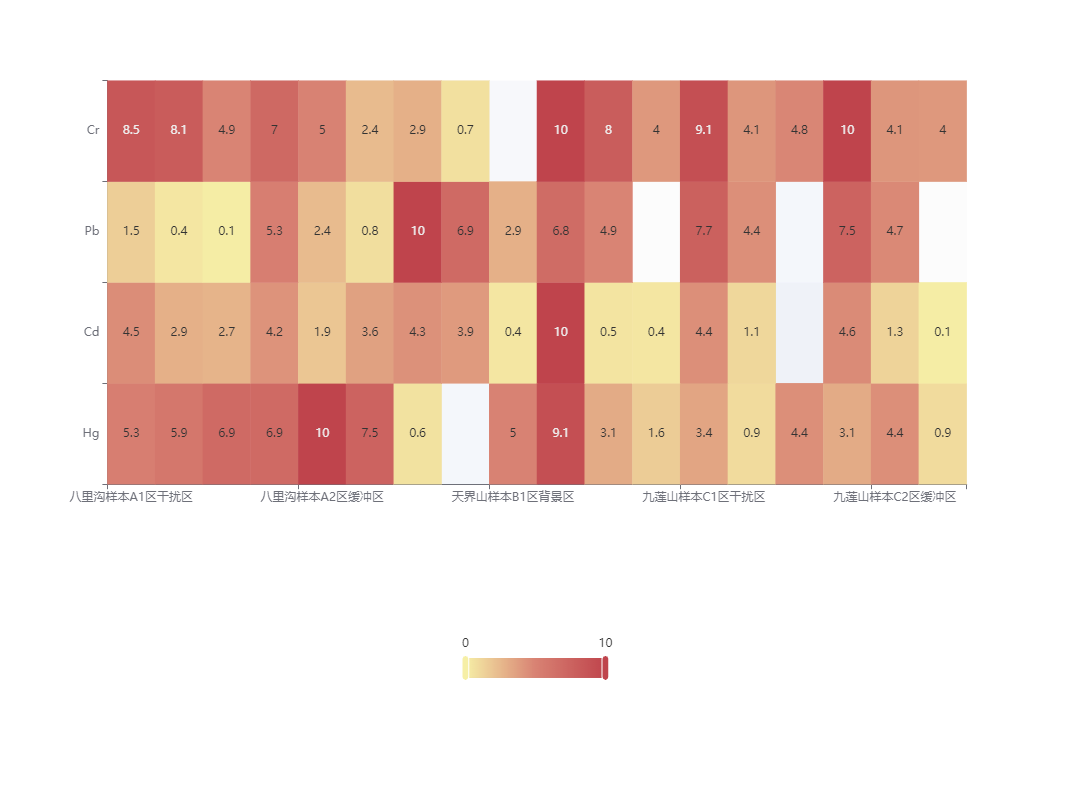

Supplement: Data S1 [file peerj-11-15780-s001.zip › Data 79893/ImageLong/ImageLong/tu4.png]

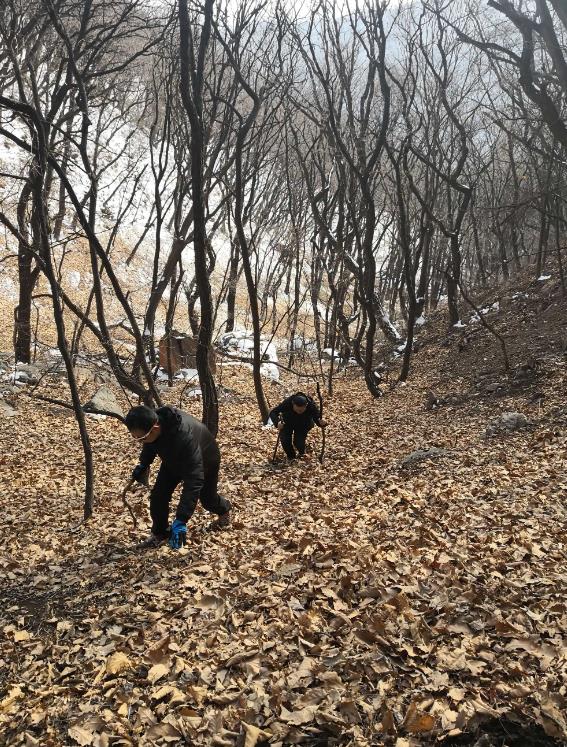

Supplement: Data S1 [file peerj-11-15780-s001.zip › Data 79893/Picture/Original 1- Scenic spot field trip pictures.jpg]

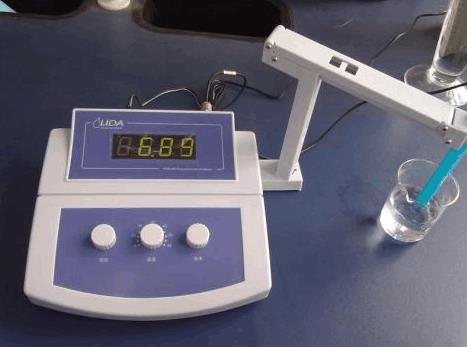

Supplement: Data S1 [file peerj-11-15780-s001.zip › Data 79893/Picture/Original 10-PH point determination.jpg]

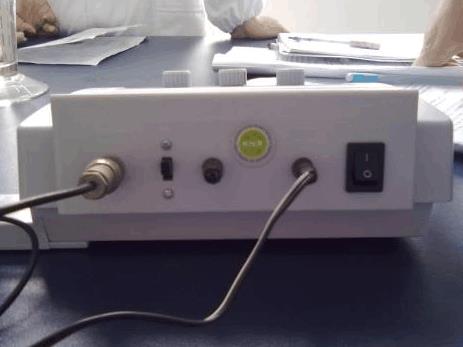

Supplement: Data S1 [file peerj-11-15780-s001.zip › Data 79893/Picture/Original 11-PH point determination.jpg]

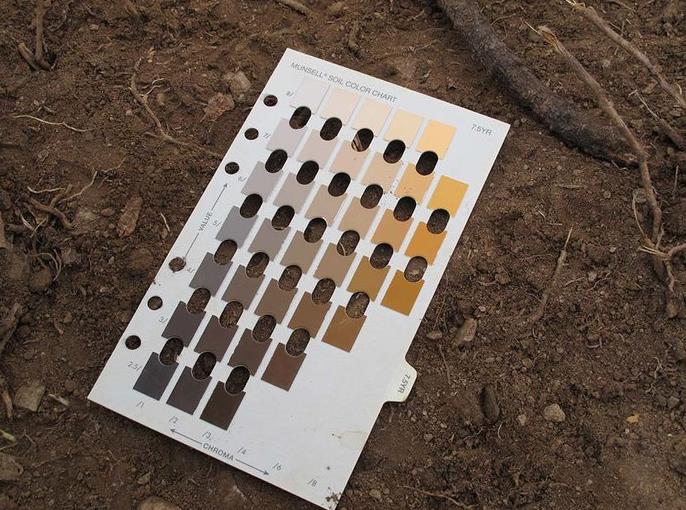

Supplement: Data S1 [file peerj-11-15780-s001.zip › Data 79893/Picture/Original 12- Soil Colorimetric card.jpg]

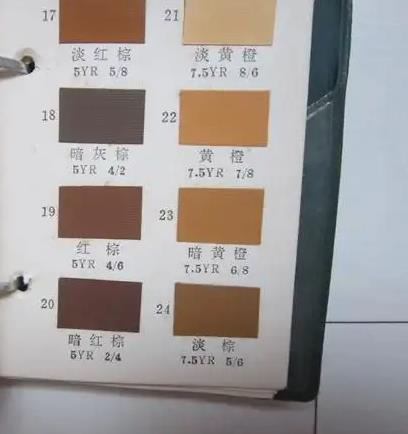

Supplement: Data S1 [file peerj-11-15780-s001.zip › Data 79893/Picture/Original 13- Soil Colorimetric card.jpg]

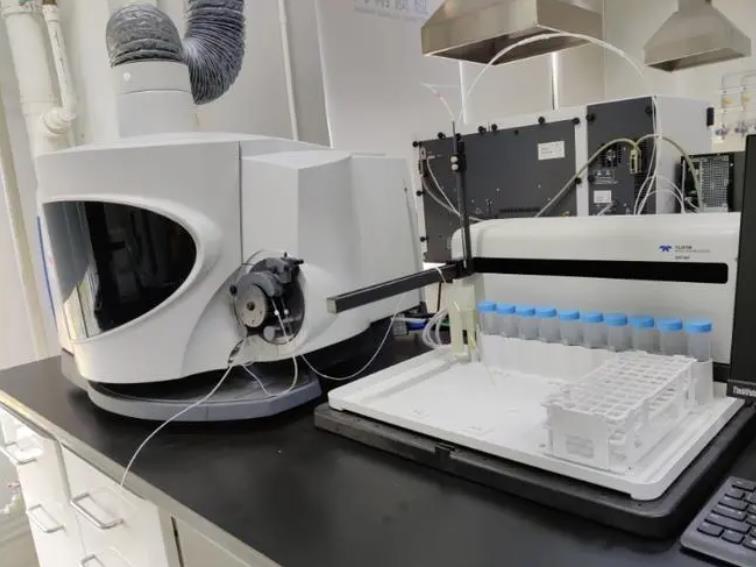

Supplement: Data S1 [file peerj-11-15780-s001.zip › Data 79893/Picture/Original 14-ICP-MS inductively coupled plasma mass spectrometer.jpg]

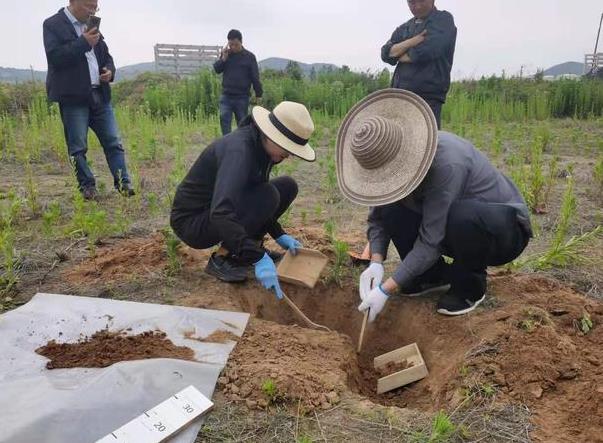

Supplement: Data S1 [file peerj-11-15780-s001.zip › Data 79893/Picture/Original 2- Scenic spot field trip pictures.jpg]

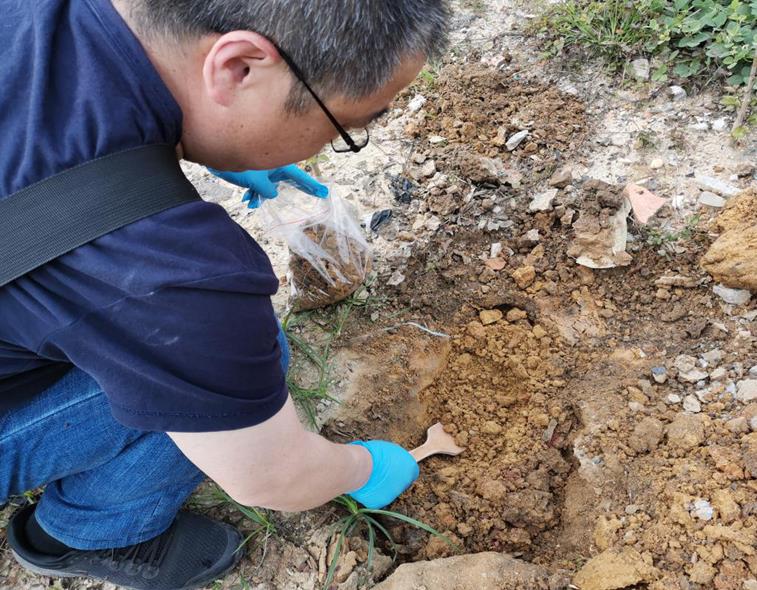

Supplement: Data S1 [file peerj-11-15780-s001.zip › Data 79893/Picture/Original 4- Scenic spot field trip pictures.jpg]

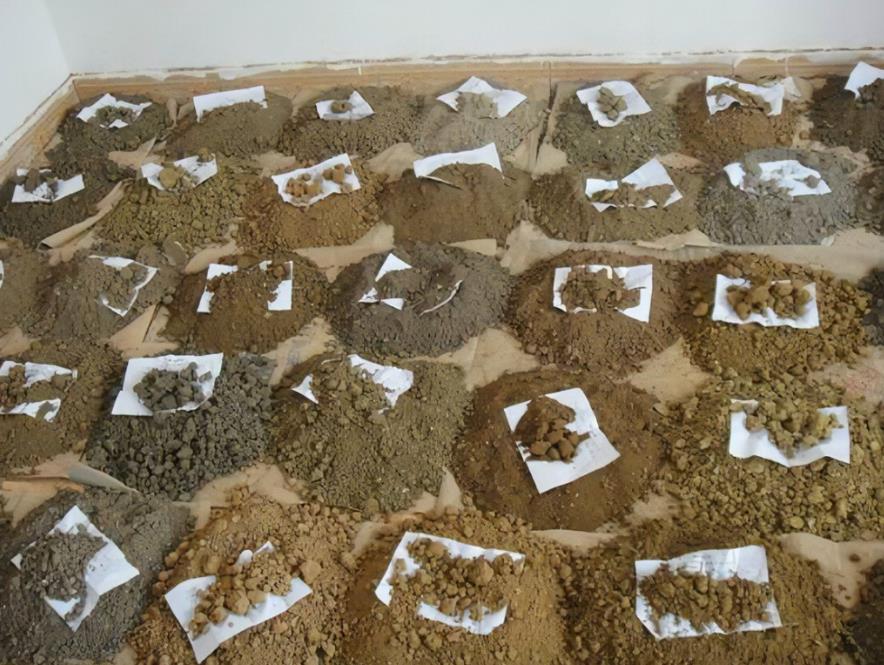

Supplement: Data S1 [file peerj-11-15780-s001.zip › Data 79893/Picture/Original 5- Soil sample processing picture.jpg]

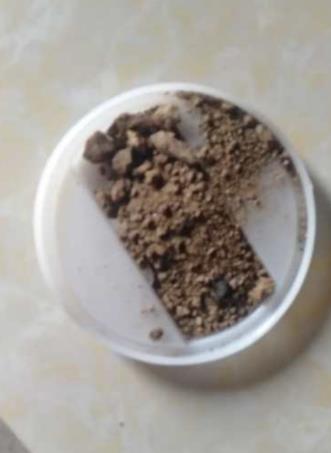

Supplement: Data S1 [file peerj-11-15780-s001.zip › Data 79893/Picture/Original 6- Picture of soil sample.jpg]

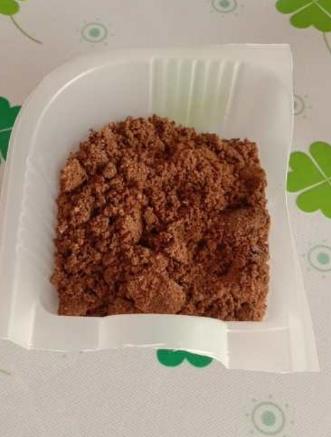

Supplement: Data S1 [file peerj-11-15780-s001.zip › Data 79893/Picture/Original 7- Picture of soil sample.jpg]

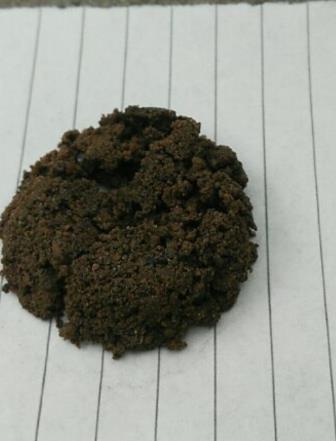

Supplement: Data S1 [file peerj-11-15780-s001.zip › Data 79893/Picture/Original 8- Picture of soil sample.jpg]

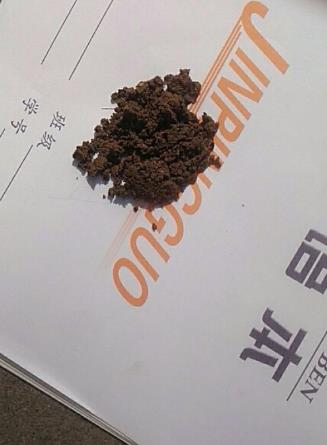

Supplement: Data S1 [file peerj-11-15780-s001.zip › Data 79893/Picture/Original 9- Picture of soil sample.jpg]

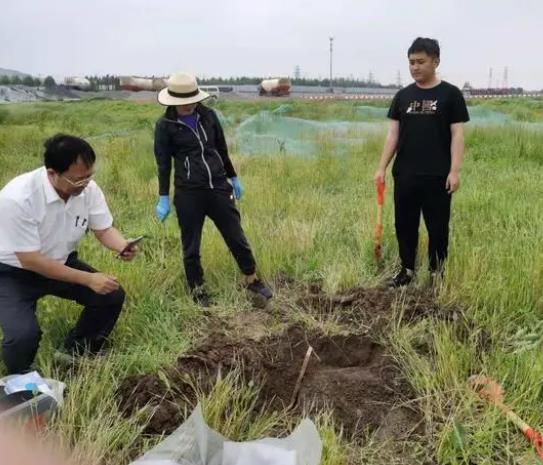

Supplement: Data S1 [file peerj-11-15780-s001.zip › Data 79893/Picture/Original3- Scenic spot field trip pictures.jpg]
